# Ewing’s Sarcoma of the Hand: An Unusual Presentation in a Young Hispanic Male

**DOI:** 10.7759/cureus.49140

**Published:** 2023-11-20

**Authors:** Dylan B McBee, Hope A Bentley, Giancarlo Toledanes

**Affiliations:** 1 School of Medicine, Baylor College of Medicine, Houston, USA; 2 School of Medicine, McGovern Medical School, Houston, USA; 3 Department of Pediatrics, Division of Pediatric Hospital Medicine, Texas Children's Hospital, Houston, USA

**Keywords:** upper extremity reconstruction, pediatrics chemotherapy, sarcoma soft tissue, limited english proficiency, extraosseous ewing's sarcoma

## Abstract

Ewing’s sarcoma is a neuroectodermal malignancy classically associated with innocuous and chronic symptomatology. Although tumors typically involve the axial skeleton, some malignancies may be confined to extraosseous tissue only. This report presents the case of a 15-year-old Hispanic male with a tender, slow-growing mass of seven months in the subcutaneous tissue of the right hand. Core needle biopsy and fine needle aspiration confirmed the diagnosis of high-grade extraosseous Ewing’s sarcoma and the patient was treated via surgical resection and chemotherapy. Nonspecific findings of Ewing’s sarcoma may mimic infection or trauma and contribute to a delay in diagnosis. However, social and economic influences including limited English proficiency and insurance status also critically affect the timing of presentation.

## Introduction

Ewing’s sarcoma is the second most common bone cancer found in the pediatric population and classically involves bones of the axial and distal skeleton [1]. Despite this, a minority of primary tumors will reliably arise in the extraosseous space [1]. Up to 20% of these cancers may present in soft tissues with reports detailing involvement of almost every major organ [1,2]. Ewing’s sarcoma of the hand is extremely rare with less than 25 cases reported to date [3-12]. A small review suggests that Ewing’s sarcoma of the hand most commonly involves the proximal phalanx of either the thumb or third digit [3]. Fatty tissue involvement at the exclusion of bone or muscle is even less likely [9].

## Case presentation

A 15-year-old Spanish-speaking, Hispanic male with no relevant past medical history presented to the emergency department with one day of acute onset right-hand pain. He reported noticing a small lump on his right hand that grew slowly over the last seven months. The lump was occasionally tender but became significantly more painful in the previous 24 hours. He described the pain as a sharp, stabbing 10/10 pain around the mass, which worsened with manipulation of his fingers. The pain was accompanied by numbness and tingling along his right arm that extended to his thumb and second and third digits. Tylenol taken at home provided little relief, prompting his visit to the emergency department.

**Figure 1:**
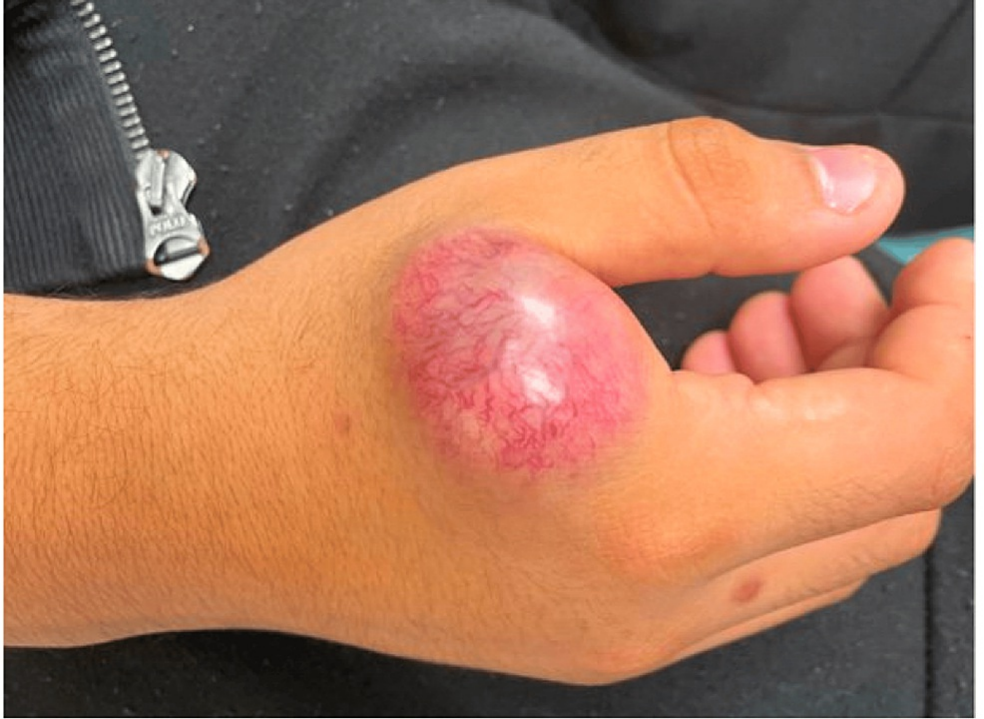
Clinical photograph of the right-hand mass

Physical examination revealed a tense, non-mobile mass involving the dorsal webspace between the first and second fingers. Overlying the mass was a pink-purple discoloration with underlying visible vasculature (Figure 1). The mass was exquisitely tender to palpation but sensation and motor function to the thumb and other digits remained intact.

**Figure 2:**
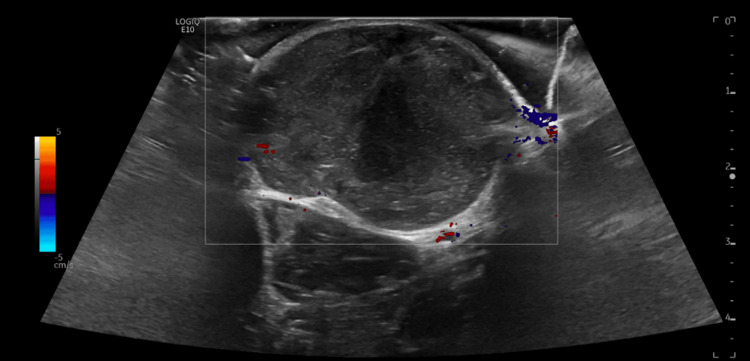
Right-hand dorsal ultrasound demonstrating heterogeneous internal echogenicity

Ultrasound revealed an oval-shaped circumscribed mass measuring ­3.2 x 1.8 x 3 cm, arising from the subcutaneous fat with large lobular margins (Figure 2). There was a complex mixed echo architecture consisting of hypoechoic and hyperechoic areas including scattered tiny hyperechoic foci. The mass also demonstrated moderate internal vascularity (Figure 2).

**Figure 3:**
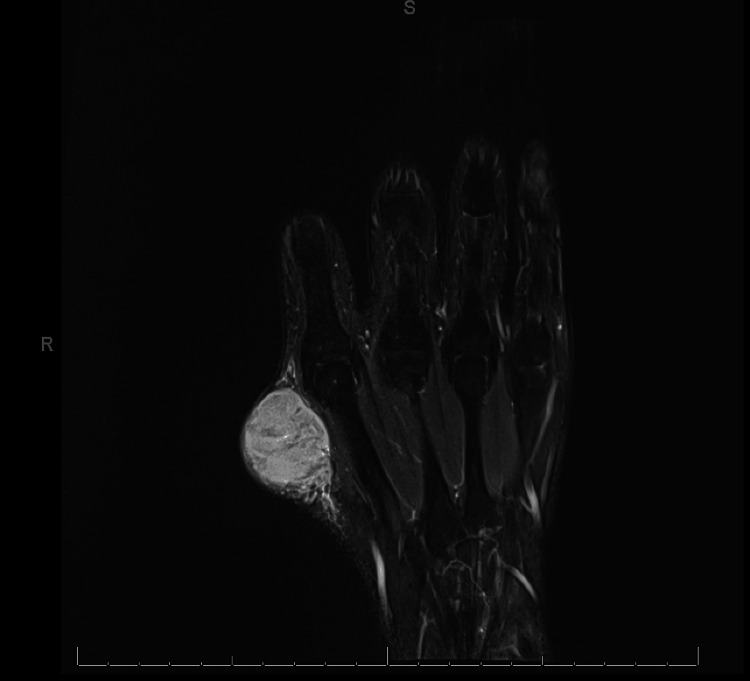
T2-weighted MRI of the right hand without contrast demonstrating heterogeneously increased T2 signal without high-flow vasculature

The patient was ultimately admitted for pain control and further evaluation of the hand mass. MRI of the right hand confirmed a well-defined mass involving only the subcutaneous fat. The mass demonstrated a heterogeneously increased T2 signal that lacked high-flow vasculature (Figure 3).

A core needle biopsy and a fine needle aspiration taken the following day confirmed the diagnosis. Smears demonstrated densely clustered small round blue cells with frequent mitoses, apoptosis, and focal necrosis consistent with high-grade features. The cells showed weak, patchy cytoplasmic reactivity for the cluster of differentiation 99 (CD99), and NKX2 nuclear staining was appreciated in 20-30% of the tumor cells. Fluorescence in-situ hybridization (FISH) demonstrated an Ewing’s sarcoma breakpoint region 1 (EWSR1) gene rearrangement; however, poor specimen preservation prevented the identification of the exact fusion partner.

Ultimately, a diagnosis of extra-skeletal Ewing’s sarcoma of the hand was made. The patient underwent complete resection of the tumor with negative margins and subsequent reconstruction using a full-thickness skin graft. Metastatic evaluation for distant disease with full body positron emission tomography (PET) and CT of the chest was negative, and the patient promptly began a standard chemotherapy regimen for non-metastatic Ewing’s sarcoma. Alternating cycles of vincristine, doxorubicin, and cyclophosphamide with high-dose ifosfamide and etoposide were administered every two weeks. To date, the patient has completed six cycles of induction chemotherapy with no significant adverse effects. Screenings for lung metastasis using chest CT continue to show no evidence of metastatic disease.

## Discussion

Although Ewing’s sarcoma is considered a pediatric cancer, it is primarily a disease of adolescence. The incidence of Ewing’s sarcoma climbs gradually with age and peaks at around 15 to 19 years of age [13]. Ewing’s sarcoma also varies according to race and ethnicity with an overwhelming majority of diagnoses made in patients of European descent [13,14]. Despite this, Hispanic patients have a higher proportion of tumors involving soft tissue only, an earlier age of onset, and ultimately inferior survival outcomes compared to white, non-Hispanic patients [14,15]. The setting and timing of the presentation may also vary according to race. One study found that Hispanic patients were more likely to present to the emergency department rather than to primary care physicians at the onset of their symptoms [14]. Hispanic patients in this study were less likely to have insurance and demonstrated a lower socioeconomic status (SES) on average compared to non-Hispanic patients [14]. Limited English proficiency (LEP) may also significantly delay the time to initial presentation among cancer patients [16]. Thus, barriers to care including lower SES and LEP play into the discrepancy seen in the presentation, prognosis, and outcomes of Hispanic patients.

Ewing’s sarcoma frequently presents to nononcologic providers with nonspecific or subtle findings. Most patients primarily report point tenderness and persistent pain upon presentation and may or may not have evidence of local erythema or swelling [1,3,13]. Clinically, Ewing’s sarcoma may mimic a localized infection or minor sports-related trauma; yet, persistent pain noticeable even at night should raise concern for an underlying malignant etiology [1,3,13]. As with our patient, the rather indolent and nonspecific findings of Ewing’s sarcoma commonly delay definitive diagnosis by several months [13]. Furthermore, a delay in care may be magnified by lower SES, which independently contributes to more advanced presentations with poorer prognosis among childhood cancers [14,17].

While radiographic and laboratory findings may support a diagnosis, histological and molecular analysis ultimately confirm the presence of Ewing’s sarcoma. For tumors involving the skeleton, alkaline phosphatase and lactate dehydrogenase may be elevated [1]. However, these findings are nonspecific and even less useful for extraosseous manifestations of the disease. Radiographic imaging of the affected bone may provide greater diagnostic value. Tumors involving bone have a cluster of cardinal features including the Codman triangle, representing new subperiosteal bone growing over the tumor, as well as a moth-eaten and onion peel appearance [1]. However, these findings are limited once again to skeletal disease. Histologically, Ewing’s sarcoma appears as densely clustered small, round, blue cells with prominent nuclei [1]. Our patient demonstrated moderate expression of CD99 on immunohistochemical staining. While not specific to Ewing’s Sarcoma, the absence of CD99 expression likely rules out this diagnosis [1,18]. NKX2.2 provides greater specificity and has gained prominence over the past decade as another standard immunohistochemical marker of this disease [19]. Genetically, Ewing’s sarcoma possesses an incredibly low mutational burden compared to other high-grade malignancies [1,13]. This is because the defining chromosomal abnormality, a translocation of EWSR1 to friend leukemia virus integration 1 (FLI1), results in a fusion protein that alone can drive malignant transformation [1,13].

The combination of cytotoxic chemotherapy and local reduction through either surgery or radiotherapy remains the mainstay of treatment with a five-year survival rate of 70% for non-metastatic, completely resected disease [1,13]. The primary prognostic factor is the presence of distant metastasis, most commonly to the lungs, bone, or bone marrow at presentation [1,13]. Thus, CT of the chest and bilateral bone marrow biopsies are typically performed at diagnosis [1,13]. Several studies now demonstrate that primary tumors involving the distal extremities reliably favor a better prognosis [1,3,5]. This advantage is likely a consequence of their relative isolation and smaller tumor size at presentation [1]. Local control with either radiotherapy or surgery is critical for successful treatment [1,13]. While not performed for our patient, neoadjuvant chemotherapy also provides the distinct advantage of early response assessment. As demonstrated in the Euro EWING 2012 clinical trial, patients should undergo a standard chemotherapy regimen involving alternating cycles of vincristine, doxorubicin, and cyclophosphamide with high-dose ifosfamide and etoposide every two weeks [20].

## Conclusions

Ewing’s sarcoma most commonly involves the axial skeleton but extraosseous presentation involving any soft tissue is possible. An indolent course defined by nonspecific findings that mimic localized infection or sports-related trauma can significantly delay diagnosis. Barriers to preventative care including lower SES and LEP may further delay early recognition of disease. Radiographic and laboratory findings offer limited value in the context of extraosseous disease while histologic and genetic studies provide true diagnostic value. Ultimately, multimodal treatment involving surgical resection and chemotherapy remains the most effective treatment for Ewing’s sarcoma localized to the distal extremities.
